# Analysing the phenotype development of soybean plants using low-cost 3D reconstruction

**DOI:** 10.1038/s41598-020-63720-2

**Published:** 2020-04-27

**Authors:** Rongsheng Zhu, Kai Sun, Zhuangzhuang Yan, Xuehui Yan, Jianglin Yu, Jia Shi, Zhenbang Hu, Hongwei Jiang, Dawei Xin, Zhanguo Zhang, Yang Li, Zhaoming Qi, Chunyan Liu, Xiaoxia Wu, Qingshan Chen

**Affiliations:** 1 College of Arts and Sciences, Northeast Agricultural University, Harbin, 150030 China; 2 College of Engineering, Northeast Agricultural University, Harbin, 150030 China; 3 College of Agricultural, Northeast Agricultural University, Harbin, 150030 China

**Keywords:** Plant breeding, Plant development

## Abstract

With the development of digital agriculture, 3D reconstruction technology has been widely used to analyse crop phenotypes. To date, most research on 3D reconstruction of field crops has been limited to analysis of population characteristics. Therefore, in this study, we propose a method based on low-cost 3D reconstruction technology to analyse the phenotype development during the whole growth period. Based on the phenotypic parameters extracted from the 3D reconstruction model, we identified the “phenotypic fingerprint” of the relevant phenotypes throughout the whole growth period of soybean plants and completed analysis of the plant growth patterns using a logistic growth model. The phenotypic fingerprint showed that, before the R3 period, the growth of the five varieties was similar. After the R5 period, the differences among the five cultivars gradually increased. This result indicates that the phenotypic fingerprint can accurately reveal the patterns of phenotypic changes. The logistic growth model of soybean plants revealed the time points of maximum growth rate of the five soybean varieties, and this information can provide a basis for developing guidelines for water and fertiliser application to crops. These findings will provide effective guidance for breeding and field management of soybean and other crops.

## Introduction

With the development of agricultural digitalisation and precision, “virtual plants” have become a hot research topic in the field of agriculture^1^. A virtual plant is the virtual visualisation of the objective plant morphological structure by 3D reconstruction, which can be done using many methods.

Virtual plants are developed by digital agriculture, computer graphics, and 3D reconstruction technology; because crop morphological structure is reproduced in 3D form, virtual plants can resolve the issue of parameter extraction caused by the low resolution of 2D images^2,3^. Virtual plants are of great significance to crop yield prediction^4^, resource and environment analysis^5^, and crop cultivation guidance^6^. Additionally, virtual plants have become extremely important for precision analysis of crop phenotypes^7,8^, analysis of plant-type characteristics^9^, computer-aided design of morphological structure^10–12^, and collaborative analysis of crop growth structure and function^13,14^.

Soybean is an important food and oil crop worldwide, and is the main source of high-quality human protein^15^. It can be predicted that, with increasing human populations and land degradation, demand for soybeans will also increase. Currently, the development of high-throughput gene sequencing technology has enabled the full discovery of genes that regulate important soybean traits^16^. However, because of the complexity of soybean plants, there have been few studies on the extraction of phenotypic parameters and analysis of growth patterns based on virtual plant models. The most common method is still the traditional manual measurement, which is destructive.

However, research on soybean plant growth morphology plays an important guiding role for analysing the growth patterns of soybean, rational seed selection, breeding, interplanting, and improving soybean yield^17^. Therefore, the accurate extraction of plant dynamic phenotypic parameters using virtual plants and the realisation of the design breeding mode, which combines molecular breeding with phenotypic breeding approaches, have become key problems to improve soybean yield.

The combination of information technology and agricultural virtual reality technology provides an important method for obtaining virtual plants. Currently, the methods used to obtain virtual plants through 3D reconstruction are roughly divided into rule-based^18^, image-based vision^19–21^, 3D scanner^22^, and 3D digitiser^23^ methods. Using a rule-based method to conduct crop 3D reconstruction and visualisation can clearly reveal crop growth patterns. However, 3D reconstruction based on 3D scanner and 3D digitiser methods has some disadvantages, such as requiring a large amount of cloud data, time-consuming processing, and expensive equipment. However, in this paper, the low-cost 3D reconstruction technology both reconstructs and performs parameter extraction of soybean plants during the whole growth period, and can also be easily used by some breeders to promote progress in crop phenotypic breeding.

In this paper, a method based on a low-cost 3D reconstruction technology is proposed to analyse the phenotypic changes of soybean during the whole growth period. The method includes two parts: 3D reconstruction and phenotypic analysis. 3D reconstruction was mainly used to obtain the morphological sequence of soybean plant images in specific growth periods with the digital image acquisition platform by circular photography; then, the 3D models of soybean plants were obtained with image-based 3D reconstruction technology. The main purpose of the phenotypic analysis was to analyse the phenotypic changes of soybean during the whole growth period by drawing a “phenotypic fingerprint” map. This method uses a combination of phenotype data and morphological model visualisation technology, which provides technical support for the design breeding mode, which combines molecular breeding with phenotypic breeding. The results will further help people understand the characteristics of soybean morphological structure, and the relationship between phenotype and field management. Precision analysis of soybean phenotypes also provides a powerful tool for field management, innovative soybean genetic breeding, and soybean plant type selection.

## Materials and Methods

### Experimental material

The field experiment was conducted at the soybean experimental base of Northeast Agricultural University of China (${{\rm{N}}44}^{^\circ }{04}^{{\prime} },{{\rm{E}}125}^{^\circ }{42}^{{\prime} }$). The soil type of the experiment was black soil. Five varieties (DN251, DN252, DN253, HN48, and HN51) were selected as experimental materials.

**Figure 1:**
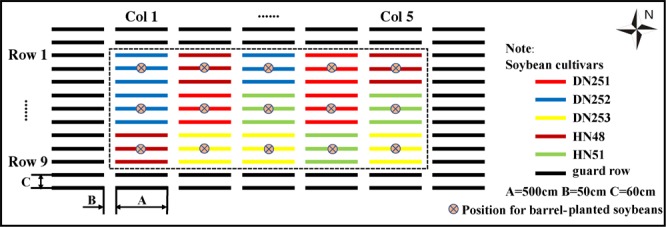
Distribution map of field experimental plots.

The experimental materials were divided into barrel and field planting materials. Barrel planting materials were planted in resin barrels with a height of 31 cm and a diameter of 27.5 cm. Multiple drainage holes were included at the bottom of the resin barrel to facilitate plant root respiration. The barrel planting materials were placed in the field environment about 20 cm underground, and the distribution location is shown in Fig. 1. According to the experimental design, a total of 15 barrels of experimental materials were obtained, and five barrels of plants were randomly selected for indoor 3D reconstruction. The field materials were planted in the test area with a ridge length of 500 cm and ridge spacing of 60 cm. Five treatments were set up by random block method. Each treatment was repeated three times, totalling 15 plots and 45 ridges. The experimental field was managed the same as the conventional field.

### Platform and methods

**Figure 2:**
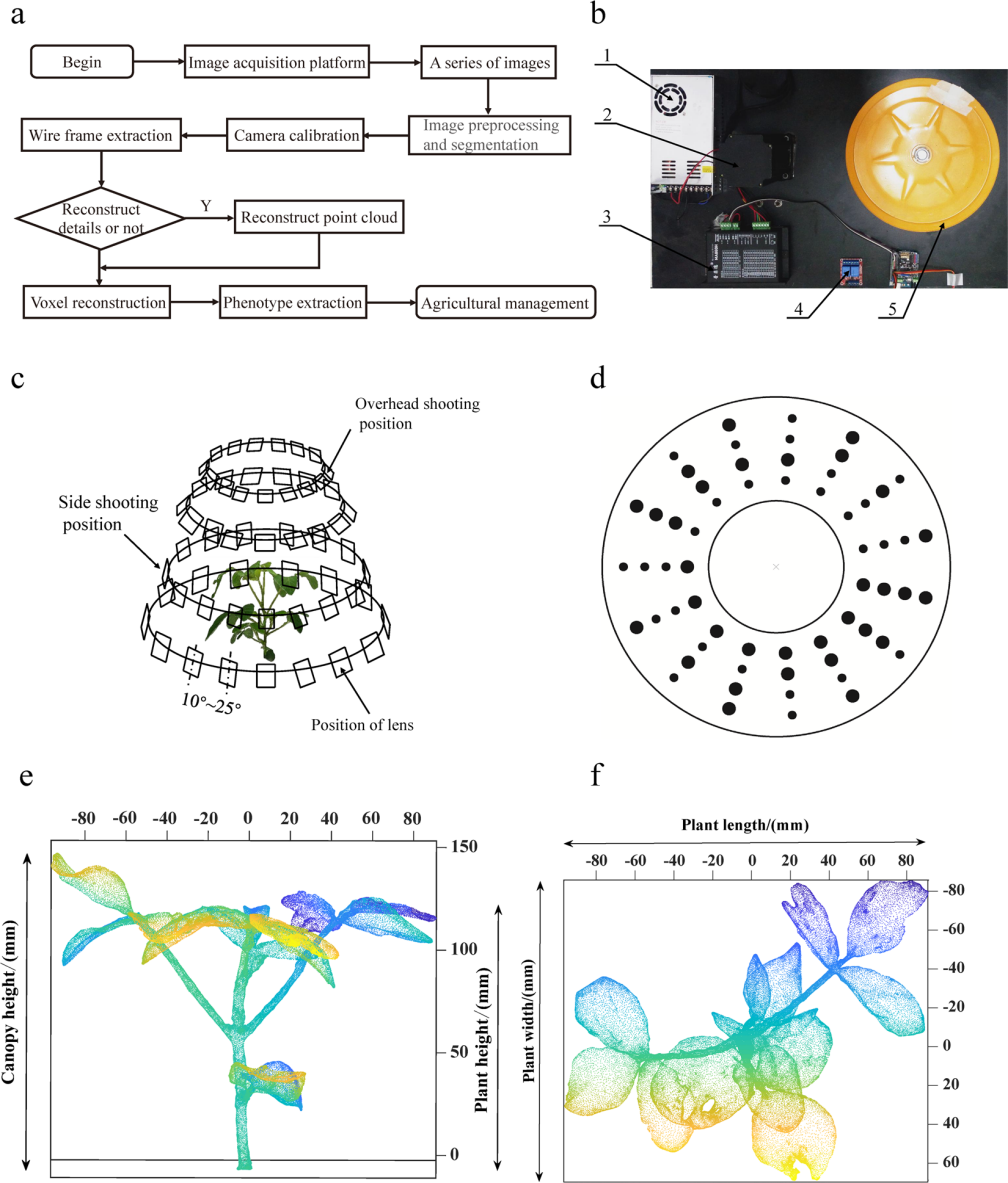
Process of soybean plant 3D reconstruction. (**a**) The technical flow chart of soybean plant 3D reconstruction. (**b**) The digital image acquisition platform (1, power supply; 2, stepping motor; 3, controller; 4, relay; 5, rotating disk). (**c**) The basic principle of multimodal photography. (**d**) The 3D reconstruction calibration template. (**e**) The main view of a 3D reconstructed soybean plant with calibrated canopy and plant height. (**f**) The overhead view with the plant length and width calibrated.

Because of the ongoing development of 3D reconstruction technology, this paper proposes 3D reconstruction and phenotype extraction technology for plants that can reconstruct plant models with 3D reconstruction technology and soybean plant morphological sequence images. The flow chart of the whole 3D reconstruction process is shown in Fig. 2a. First, the morphological sequence images of soybean plants during specific growth periods were obtained through the digital image acquisition platform. Then, the morphological sequence images were preprocessed. Finally, 3D reconstruction was performed by camera calibration, feature extraction; then, the corresponding phenotypic parameter extraction and application were completed.

#### Digital image acquisition platform

The digital image acquisition platform was built on the principle of multi-vision stereo vision^24^, and was mainly composed of a digital camera, rotary table, servo stepper motors, lead-straight sliding rail, sensors, a control panel, supplementary light, and background cloth (Fig. 2b).

**Figure 3:**
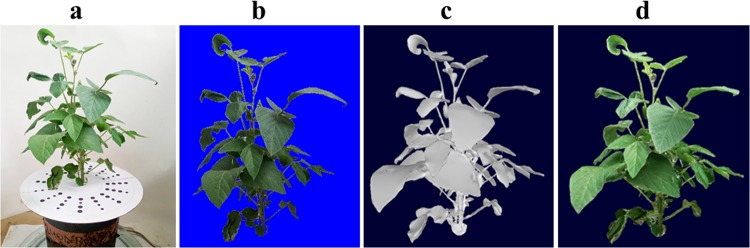
A 3D reconstructed model of soybean DN252 during the whole growth period. (**a**) The original image obtained through the digital image acquisition platform. (**b**) The image obtained by image preprocessing. (**c**) The result of 3D reconstruction. (**d**) A 3D model with colour texture.

Two stepper motors drive the rotary table to rotate automatically through the synchronous belt, and the other stepper motor controls the height of the digital camera on the lead-straight slide rail to adapt to different plant heights in different growth periods. The main working principle of the platform is to construct a multi-view stereo vision system with digital cameras at different angles. Using circular photography, the platform takes photos of the target plants in the range of 10°–25°, and combines them with an automatic turntable and calibration pad (Fig. 2c). Sixty photos can be obtained through four groups of circular rotations shooting at different angles, and the original image obtained by the digital image acquisition platform is shown in Fig. 3a. This method effectively improves the problem of mutual occlusion between soybean leaves; then, the morphological sequence images of the target plant are obtained for 3D reconstruction^25^.

#### Digital image preprocessing

The image quality obtained by the digital image acquisition platform directly determines the 3D reconstruction accuracy. However, during image shooting and transmission, image quality is often affected by various noise sources because of factors associated with artificial shooting and equipment, and this has a negative impact on the subsequent image processing (such as image recognition and segmentation) and camera calibration if the signal-to-noise ratio of the image is large^26^.

Therefore, to improve the accuracy of camera calibration and 3D reconstruction, it is necessary to denoise the image. Filtering and smoothing are used to eliminate image noise. Based on analysis of the actual soybean plant morphological sequence images, it was concluded that most of the image pollution noise was Gaussian white noise. Therefore, the threshold denoising method based on wavelet transform was selected to denoise the soybean plant morphological sequence images^27^. The background segmentation of the image was identified using the blue screen matching technology^28^. The plant image obtained using the preprocessing method is shown in Fig. 3b.

### Camera calibration

The parameters for calibration differ among different cameras. Therefore, it is very important to select a calibration template that can improve accuracy and robustness. In this study, a calibration template generated by 3D software object modeler^29^ was selected for camera calibration. The calibration template was composed of 15 sets of patterns, each of which contained four points and was arranged in a large radial circle (Fig. 2d). The points on the calibration template are easily recognised, which prevents the more complex calculation problems of multi-frame structure motion technology^30^.

Camera calibration is a basic and key step in the early stage of 3D reconstruction that can lays the foundation for stereo image matching during 3D reconstruction. Camera calibration is used to determine the status of the internal structure (the internal parameters) and the relationship between the camera and the location of the scene (the external parameters) when the camera takes pictures. The accuracy of parameters directly affects 3D reconstruction accuracy^31^.

In the classical pinhole camera model, the relationship between the points in the image and the actual coordinates can be represented by the world coordinate, camera coordinate, image coordinate, and pixel coordinate systems. Any point in space and its projection on the camera image plane must meet the following projection formula:

1 $${\boldsymbol{s}}\cdot {\boldsymbol{m}}={\boldsymbol{K}}[{\boldsymbol{R}}\cdot {\boldsymbol{t}}]{\boldsymbol{M}}$$

In the formula, ***s*** is any scale factor and ***m***(*u*, *v*, 1)^*T*^ is the coordinate of point $n$ in the image coordinate system. ***M***(*x*, *y*, *z*,1)^*T*^ is the coordinate of point *n* in the world coordinate system. [***R*** · ***t***] is the camera external parameter matrix. ${\boldsymbol{R}}$ an ${\boldsymbol{t}}$ are, respectively, the rotation matrix and conversion parameters in the process of transformation from world coordinate system to camera coordinate system. ${\boldsymbol{K}}$ is the internal parameter matrix of the camera, which mainly includes $fx$, .., $u0$, $v0$, $fx$, and $fy$, which are units of pixel length. The expression $$K=[\begin{array}{ccc}fx & 0 & u0\\ 0 & fy & v0\\ 0 & 0 & 1\end{array}]$$. $({u}_{0},{v}_{0})$ is the coordinate of the principal point.

However, in practical applications, the lens will show some geometric distortion. The existence of image geometric distortion both affects the visual effect of the image and also increases the difficulty of subsequent image processing; therefore, it is very important to select a suitable distortion model^32^. The most common method is decomposing geometric distortion into radial distortion, tangential distortion, and prism components. The correction model of lens distortion is as follows:

2 $${\delta }_{x}(x,y)=({k}_{1}x({x}^{2}+{y}^{2})+{k}_{2}x{({x}^{2}+{y}^{2})}^{2})+({p}_{1}(3{x}^{2}+{y}^{2})+2{p}_{2}xy)+{s}_{1}({x}^{2}+{y}^{2})$$

3 $${\delta }_{y}(x,y)=({k}_{1}y({x}^{2}+{y}^{2})+{k}_{2}y{({x}^{2}+{y}^{2})}^{2})+({p}_{2}(3{x}^{2}+{y}^{2})+2{p}_{1}xy)+{s}_{2}({x}^{2}+{y}^{2})$$

4 $${\delta }_{x}(x,y)={x}_{u}-x$$

5 $${\delta }_{{\rm{y}}}(x,y)={y}_{u}-y$$

In formulas (2) and (3), ${\delta }_{x}(x,y)$ and ${\delta }_{{\rm{y}}}(x,y)$ represent the distortion values, respectively. The first part is radial distortion, the second part is centrifugal distortion, and the third part is thin prism distortion. ${k}_{1},{k}_{2},{p}_{1},{p}_{2},{s}_{1}$ and $s2$ are the distortion coefficients. However, it has been shown that the total error of non-radial distortion is only 1/7–1/8 of that of radial distortion^33^. Tsai also proposed that tangential distortion can be neglected for most computer vision applications^34^.

Therefore, the radial distortion correction model is as follows:

6 $${u}_{d}=u+{k}_{1}(u-{u}_{0}){r}^{2}$$

7 $${v}_{d}=v+{k}_{1}(v-{v}_{0}){r}^{2}$$

8 $${r}^{2}={x}_{n}^{2}+{y}_{n}^{2}$$

In the formula, $({x}_{n},{y}_{n})$ is the idealised coordinate, $(u,v)$ is the corrected coordinate, $({u}_{d},{v}_{d})$ is the image point with radial distortion, and ${k}_{1}$ is the radial distortion coefficient.

In this study, a precision estimation method of camera parameters based on the RANSAC algorithm was adopted. This method has strong robustness under the conditions of substantial noise and incorrect point matches. The RANSAC algorithm, proposed by Fishler and Bolles in 1981, is simple but powerful technology that is widely used in the field of computer vision for model parameter estimation tasks^35^, such as camera parameter estimation^36,37^, motion estimation^38,39^, image registration^40,41^, projection reconstruction^42^, and ellipse extraction^43^. During camera calibration, 15 photos were taken for each group of calibration templates (with and without plants). Then, the camera was calibrated based on the RANSAC calibration algorithm proposed by Lv^44^ and Zhou^45^. Finally, the corresponding internal and external parameter matrices of the camera at different angles were obtained by detecting the feature points on the calibration template.

### reconstruction method

For 3D reconstruction, feature point detection and stereo feature matching are important steps. In this study, the SURF algorithm was used to extract image features and the RANSAC algorithm was used to achieve stereo feature point matching; then, a 3D point cloud model was obtained^46^. Because the surface reconstruction method based on the Poisson equation combines the advantages of global fitting surface reconstruction methods and local fitting surface reconstruction methods, it has better elasticity for data noise processing. Therefore, in this study, a surface reconstruction method based on the Poisson equation was used to more accurately obtain and optimise corner points, and find the matching points of corner features. Consequently, the precise surface of the reconstructed object was obtained^47,48^.

The steps of Poisson surface reconstruction are as follows:

### Define octree

When the initial 3D point cloud of a soybean plant was obtained from the soybean plant pictures, it was necessary to reconstruct the surface of the 3D point cloud. In this study, octree structure was adopted to infer the spatial description of the 3D point cloud of soybean plants. From the initial node, each node extended into eight sub-nodes, and the corresponding spatial cube of the node was divided into eight parts; this was conducted until the number of layers specified in the 3D reconstruction was satisfied. The root node of the initial 3D point cloud in this paper was a boundary box around the surface that was reconstructed^49,50^. Therefore, octree was used to divide point clouds, and the data structure of each layer of octree nodes was established.

### Set the space function

The Poisson equation is expressed as follows:

9 $${\nabla }^{2}\varphi =f$$

In the formula, $F$ is a real or complex equation in space. In this paper, $f$ refers to the 3D surface of soybean plants. If the $f(x)$ of a point is equal to 0, then the point is on the surface; if $f(x) > 0$ or $f(x) < 0$, then the point is not on the surface.

The basic purpose of the Poisson equation is to solve implicit function $\varphi$ based on the domain A and vector field $\overrightarrow{V}$. The solution is used to make an optimal gradient of the implicit function approximate to the vector field $\overrightarrow{V}$ as follows:

10 $$\min \,{{\int }_{A}\Vert \nabla \varphi -\overrightarrow{V}\Vert }^{2}dA$$

Therefore, the main task of 3D reconstruction of soybean plants based on Poisson equation is to accurately calculate its gradient based on the sample points in the point cloud to approximate the implicit function of vector field $\overrightarrow{V}$ as accurately as possible. Consequently, the Poisson equation was solved as follows:

11 $$\nabla \varphi =\overrightarrow{V}$$

### Create a vector field

For each node $o\in O$ in the octree, $o$ here refers to the node in the octree structure, and $O$ refers to the octree. Therefore, the node function equation can be defined as:

12 $$Fo\equiv F(\frac{q-o\cdot c}{o\cdot w})\frac{1}{o\cdot {w}^{3}}$$

In the formula, $o\cdot c$ refers to the centre of node $o$, and $o\cdot w$ refers to the width of node $o$.

The basis function $F$ can be obtained by n-dimensional convolution of formula (13). The larger the value, the better the reconstruction effect. However, the time and complexity of the algorithm correspondingly increase:13

The expression of the basis function *F* is as follows:

14 $$F(x,y,z)={(B(x)B(y)B(z))}^{\ast n}$$

Point cloud data obtained based on multiple superpositions of the Poisson equation tend to be inconsistent in density and dispersion. Therefore, in this study, the weighted coefficient of the nearest neighbour node was used for calculation. Using this method, the precision of high point cloud data can be improved, and the approximate vector of the indicated function gradient field can be defined as:

15 $$\overrightarrow{V}(q)\equiv \sum _{s\in S}\sum _{o\in NgbrD(s)}{\alpha }_{o,s}Fo(q)s.\overrightarrow{N}$$

$NgbrD(s)$ is the eight nodes with depth D, and is the weighted coefficient of interpolation. Based on the requirement of the second-order smoothness of soybean plants in the process of 3D reconstruction, the method of cubic spline interpolation was used to calculate the weighted coefficient.

### Solve the poisson equation

There are many methods that are used to solve the Poisson equation. In this paper, the Gauss–Seidel iteration method was selected to solve the Poisson equation^47^.

If the coefficient matrix $A$ of system $Ax=b$ is non-singular and $aii\ne 0(i=1,2,\cdots ,n)$, then:

$${\rm{D}}=(\begin{array}{cccc}a11 & 0 & \cdots  & 0\\ 0 & a21 & \cdots  & 0\\ \cdots  & \cdots  & \ddots  & \cdots \\ 0 & 0 & \cdots  & ann\end{array})\,L=(\begin{array}{cccc}0 & 0 & \cdots  & 0\\ a21 & 0 & \cdots  & 0\\ \cdots  & \cdots  & \cdots  & \cdots \\ an1 & an2 & \cdots  & 0\end{array})\,{\rm{U}}=(\begin{array}{cccc}0 & a12 & \cdots  & a1n\\ 0 & 0 & \cdots  & a2n\\ \cdots  & \cdots  & \cdots  & \cdots \\ 0 & 0 & \cdots  & 0\end{array})$$

The formula of the Gauss–Seidel iteration method can be expressed in the following form if $A$ is divided into $A=D-L-U$ and each of $D,L,U$ are similar to Jacobian iteration:

16 $${X}^{(i+1)}={D}^{-1}(L{X}^{(i+1)}+U{X}^{(i)}+b),\,(i=0,1,\cdots )$$

Then, both sides are multiplied by matrix $D$:

17 $$(D-L){X}^{(i+1)}=U{X}^{(i)}+b,\,(i=0,1,{\boldsymbol{\cdot }}{\boldsymbol{\cdot }}{\boldsymbol{\cdot }}{\boldsymbol{)}}$$

Finally, both sides are multiplied by matrix ${(D-L)}^{-1}$:

18 $${X}^{(i+1)}={(D-L)}^{-1}U{X}^{(i)}+{(D-L)}^{-1}b,\,(i=0,1,{\boldsymbol{\cdot }}{\boldsymbol{\cdot }}{\boldsymbol{\cdot }}{\boldsymbol{)}}$$

In the formula, ${(D-L)}^{-1}U$ is called the Gauss–Seidel matrix.

### Extract the isosurface

If the equation of a surface function in space is $F(x,y,z)$, then the isosurface equation needed in the process of 3D reconstruction based on Poisson’s equation is $F(x,y,z)=V$. This equation represents the surface when the space surface function $F(x,y,z)$ is equal to a given value (that is, the surface is composed of $\varOmega =\{(x,y,z):F(x,y,z)=V\}$).

In this experiment, the moving tetrahedron algorithm, which is similar to the moving cube method, was used. This method can effectively avoid the shortcomings of the moving cube method, and more accurately reflects the detailed texture of soybean plants^51^. The soybean plants reconstructed based on the Poisson equation are shown in Fig. 3c–d.

### Extraction and analysis of dynamic plant phenotypes

#### Definition and extraction of dynamic plant phenotypes based on 3D models

The extraction of dynamic phenotypic parameters of soybean plants based on 3D models is key for researching morphological changes of soybean plants. In this study, the parameters of soybean plant length, plant width, plant height, canopy height, canopy area, plant volume, and plant type were calibrated according to Qiu^52^.

Soybean plant height was defined as the distance from cotyledon node to growing point, and canopy height was the distance from cotyledon node to the highest point of the plant (Fig. 2e). The minimum circumscribed rectangle of the ground projection of the 3D model canopy is shown in Fig. 2f. The length of the minimum circumscribed rectangle was defined as the plant length and the width as the plant width. The product of plant length and width was defined as the canopy area. The product of plant width, plant length, and canopy height was defined as the plant volume.

The phenotypes of the plant 3D point cloud model, which included plant height, plant length, plant width, and canopy height, can be automatically obtained by the point cloud classin point cloud tools for MATLAB (https://www.geo.tuwien.ac.at/downloads/pg/pctools/publish/pointCloudSelect/html/pointCloudSelect.html) and pcshow function in MATLAB.

#### Plant phenotype analysis based on 3D models

To improve the accuracy of the 3D reconstruction technology, the same standards were adopted to manually measure the plant. The Pearson correlation analysis was used to analyse the accuracy of the parameters plant length, plant width, canopy height, plant height, canopy area, and plant volume. Simultaneously, the radar charts of the plants were drawn according to the phenotypic parameters, and quantitative analysis of the phenotypic parameters was carried out.

Because the phenotypic parameters of soybean during the whole growth period can be obtained based on this method, the plant growth model and growth rate model can be analysed by logistic model. Compared with other parametric growth models, a logistic growth curve analysis was conducted using a 3-parametric model in this study^53^. The parameter estimations of the logistic model (Eq. 19) were implemented by gradient descent (sgd package and glm function based on R language)^54^. The specific analysis formula was as follows:

19 $$N)(t)=\frac{K}{1+(\frac{K}{{N}_{0}}-1)\,{e}^{-rt}}$$

In the formula, N(t) refers to the value of target phenotype at time t, t refers to the days after seedling emergence, N_0_ refers to the initial value of target phenotype, K refers to the environmental capacity and is also the level asymptotic value of target traits, and *r* refers to the maximal growth rate.

The growth rate model can be used to analyse the growth ability of crops, and can also help analyse the growth of crops under increasing population sizes and promote precision management of a certain variety of crops in the field.

The growth rate analysis model of soybean based on the logistic growth curve is as follows:

20 $$\{\begin{array}{l}\frac{dN}{dt}=r(1-\frac{N}{K})\,N\\ N({t}_{0})={N}_{0}\end{array}$$

When $N(t)=\frac{K}{2}$, the growth rate has reached the maximum. Consequently, the time to reach the maximum growth rate can be accurately calculated.

## Results

### Morphological changes of soybean plants based on 3D reconstruction models

In this study, 3D morphological reconstruction of DN251, DN252, DN253, HN48, and HN51 soybean cultivars in the growth periods of V3, V4, R1, R2, R3, R5, R6, R7, and R8 were inferred by low-cost 3D reconstruction technology.

**Figure 4:**
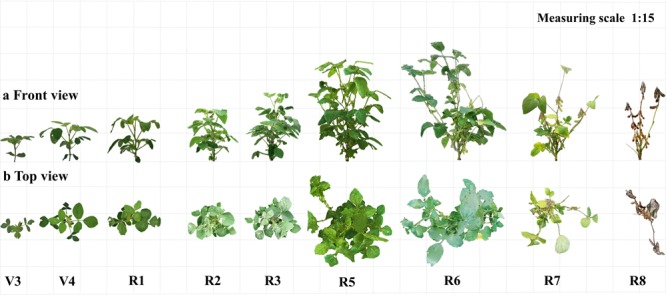
Morphological changes of soybean plants during the different growth and development stages based on the 3D model of the DN252 plant. (**a**) The front view. (**b**) The top view. V3, V4, R1, R2, R3, R5, R6, R7, and R8 refer to the stages in sequence^55^.

Figure 4 shows the main view (Fig. 4a) and top view (Fig. 4b) of the 3D reconstruction models of the soybean DN252 plant. This clearly reflects the whole process of soybean development. Figure 4 shows that the 3D models obtained based on the low-cost 3D reconstruction technology truly reflected the colour and texture characteristics of soybean plants. Therefore, this method facilitates high-quality 3D reconstruction and morphological visualisation of soybean plants and can more realistically reveal the morphology and growth state of plants.

### Accuracy analysis of 3D models

#### Comparison of plant phenotypes between field- and barrel-planted soybean

**Figure 5:**
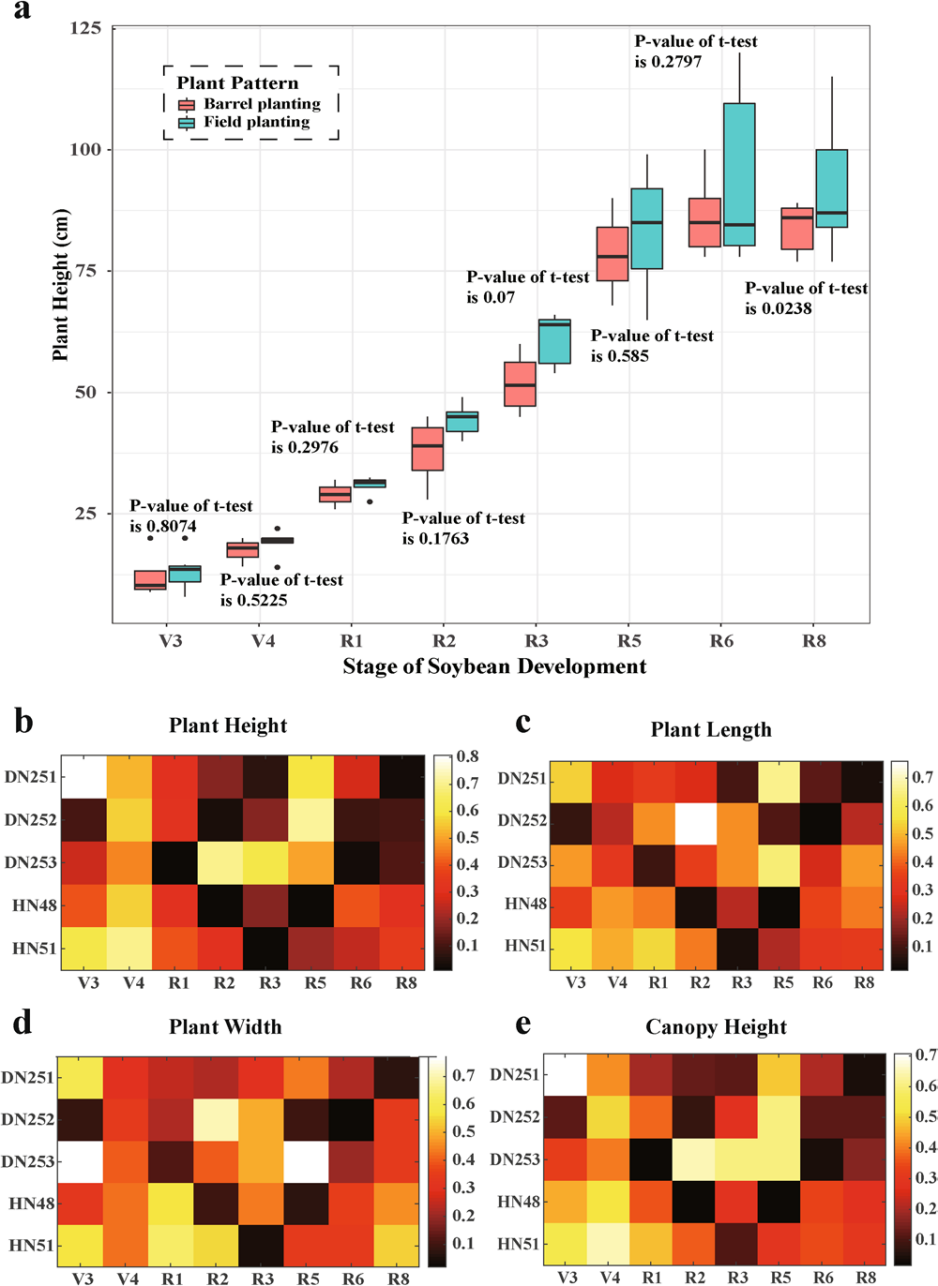
Comparison of plant phenotypes between field- and barrel-planted soybean. (**a**) A box plot of DN251 plant height in different planting patterns (field- or barrel-planted); the value above or below each group refers to the p-value of the t-test between field- and barrel-planted phenotypes. b–e depict heat maps of plant height, plant length, plant width, and canopy height, respectively. The colour of each square in every heat map denotes the p-value of the t-test between the field- and barrel-planted plants for the corresponding phenotype.

In this study, three barrels of each variety were planted and scattered in the middle of their respective test areas (see Fig. 1). These materials were used to explain whether there were significant differences between the phenotypes of barrel plants and those of other plants in the field. Figure 5a depicts the box plot and t test results of plant height differences between barrel and field planting of DN251; although the plant height of barrel-planted plants was significantly lower than that of the field-planted plants at each stage of soybean development, the overall difference was not significant. This conclusion was verified by the subsequent t-test; that is, there was no significant difference in plant height between barrel and field planting.

This result was also verified for plant height phenotype of other varieties (Fig. 5b). Analysis of differences between plant length (Fig. 5c), plant width (Fig. 5d), and canopy height (Fig. 5e) of barrel- and field-planted plants was also similar to that of plant height (in this analysis, when alpha is 0.1, the difference may be significant, but the probability of this occurring is very low). It was concluded that there was no significant difference in plant height, plant length, plant width, and canopy height between the barrel- and field-planted plants.

We studied and observed the growth of the plants in the field with the phenotype obtained by the barrel-planting method after treatment (porous bottom and the barrels buried 20 cm below ground). Therefore, it was possible to study and observe the growth of field plants using the phenotypic data obtained from the barrel-planted plants (multiple drainage holes at the bottom and the barrels buried 20 cm below ground).

#### Accuracy analysis of 3D models

**Figure 6:**
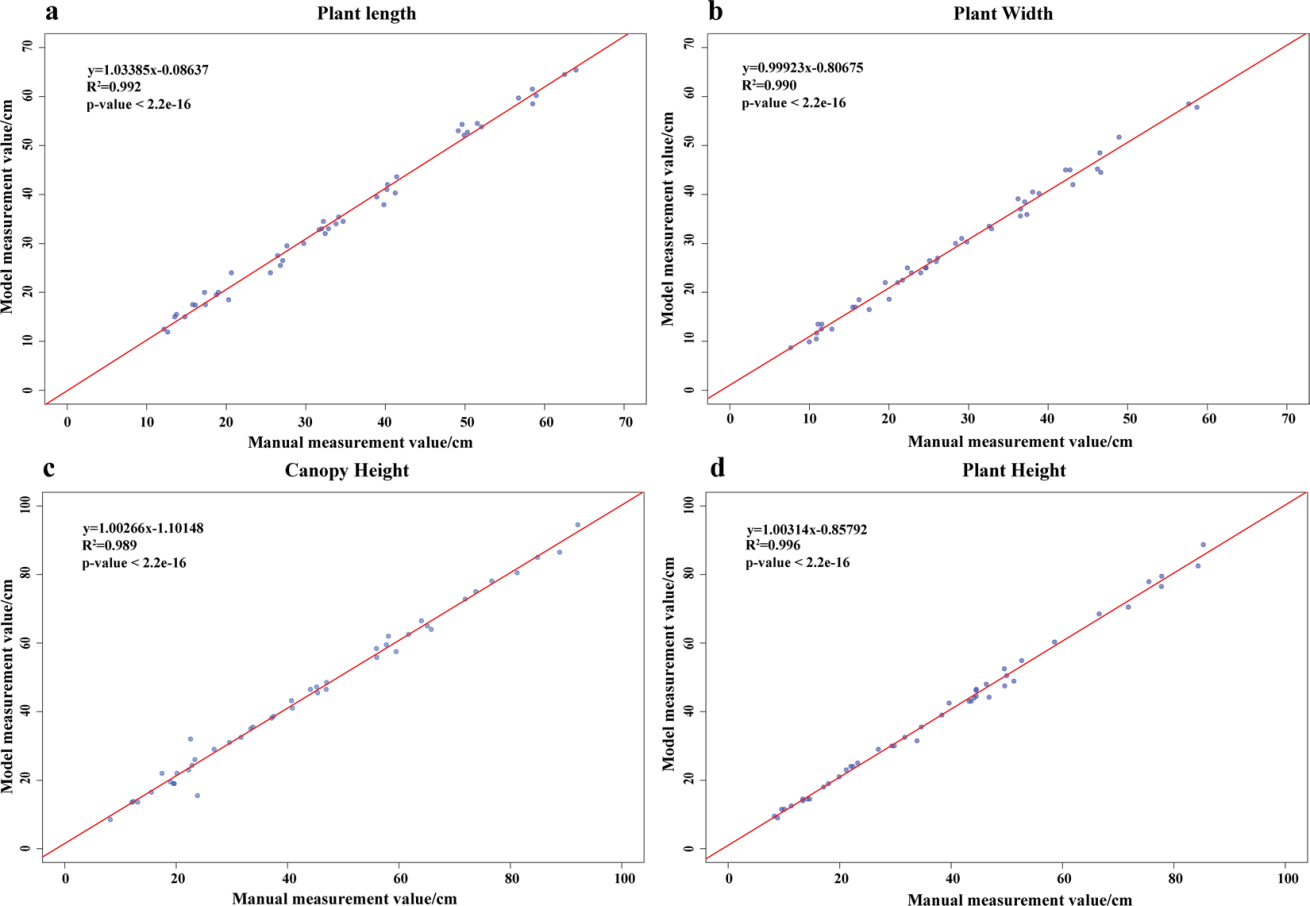
Accuracy analysis of phenotype extraction values based on 3D model and manual measurement values. a–d, respectively, represent accuracy analysis of plant length, plant width, canopy height, and plant height phenotypic quantification values based on 3D model and manual measurement values, of which the sample size N of the four phenotypic indicators was 45.

To verify the accuracy of the 3D models obtained by our method, correlation analysis was conducted between the plant length, plant width, canopy height, and plant height extracted based on DN252 3D models and the manually measured values. The Pearson correlation coefficients were used to evaluate the reconstruction effect (Fig. 6). The correlation coefficients of plant length, plant width, canopy height, and plant height extracted from the models with the manually measured values were 0.992 (Fig. 6a), 0.990 (Fig. 6b), 0.989 (Fig. 6c), and 0.996 (Fig. 6d), respectively. In summary, the correlation coefficients were higher than 0.98, which showed that the accuracy of the models obtained by 3D reconstruction based on morphological sequence images was higher. Even for soybean plants with complex growth structure around the R5 stage, morphological visualisation and parameter extraction can be effectively inferred. Therefore, the data extracted based on this method can be fully used for plant phenotype analysis.

### Analysis of dynamic phenotype changes of soybean plants based on 3D models

#### “Phenotypic fingerprint” analysis of soybean plants based on 3D models

**Figure 7:**
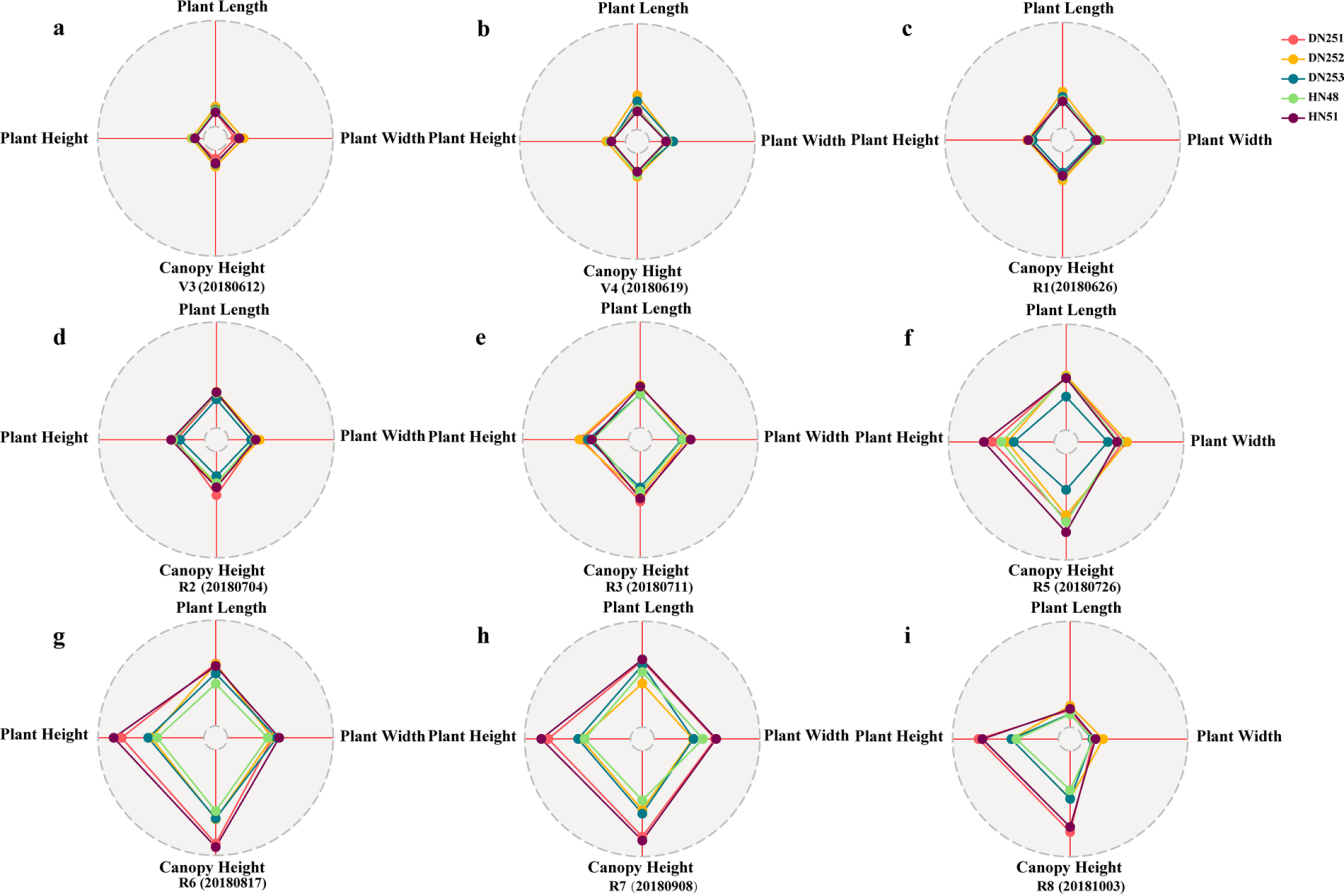
Radar charts of soybean plants based on growth and development stages. Each radar chart is made up of four features (plant height, plant length, plant width, and canopy height) of the five candidate soybean varieties (DN251, DN252, DN253, HN48, and HN51). (**a–f**) Phenotypic fingerprints of soybean plants based on different growth and development stages. Each sub-graph may be called a growth stage phenotypic fingerprint.

**Figure 8:**
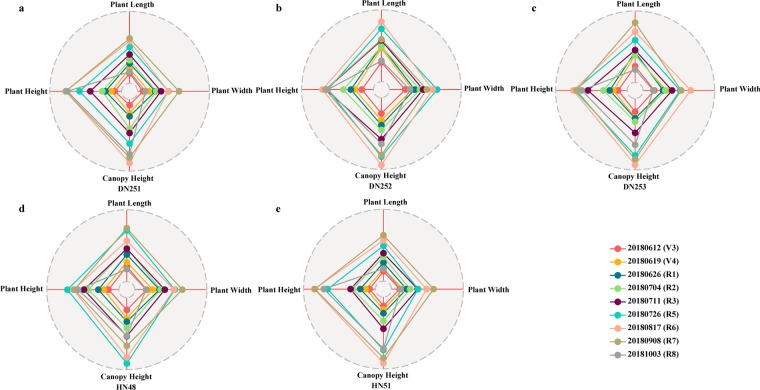
Radar charts of plants based on soybean varieties. Each radar chart is made up of four features (plant height, plant length, plant width, and canopy height) and nine growth and development stages in sequence (V3, V4, R1, R2, R3, R5, R6, R7, and R8). (**a–e**) Phenotypic fingerprints of soybean plants based on different soybean varieties (DN251, DN252, DN253, HN48, and HN51). Each sub-graph may be called a variety phenotypic fingerprint.

Phenotypic parameters obtained from the 3D reconstruction models can be used to draw radar charts, which reflect plant morphological changes (Figs. 7, 8). As each sub-graph looks like a human’s fingerprint, they are called “phenotypic fingerprints”.

The concept of a phenotypic fingerprint is new for phenotypic analysis and phenotypic breeding. The greatest advantage of phenotypic fingerprinting is that it integrates and displays multi-dimensional data, and accurately reveals quantitative phenotypic variation with internal connections. Moreover, phenotypic fingerprinting can also be used for plant morphological structure analysis and plant type selection. Consequently, this approach can provide data support for breeding, cultivation, resource, and environment analysis, especially for quantitative trait locus research. The phenotypic data extracted from the 3D models can be seen in Supplementary File 1.

Phenotypic fingerprint drawings (Figs. 7–8) were constructed for the five soybean varieties. Figure 7 is the phenotypic fingerprint formed by the sequence of the growth periods and can be used to analyse the differences of phenotypic parameters of different soybean varieties in the same period. In the V3, V4, R1, R2, and R3 periods, the growth of plant length, plant width, canopy height, and plant height of the five soybean varieties were similar (Fig. 7a–e). However, from the beginning of the R5 period, the differences of growth among the five cultivars gradually increased. Among the four traits, plant height and canopy height grew faster than plant length and plant width (Fig. 7f). In the R6 and R7 periods, the growth differences of the five varieties almost reached the maximum. The R6 and R7 stages represent the full seed period, and most nutrients have been taken up by the time the plant reaches the R6 stage. Therefore, these growth differences may be a target for the field management of different varieties, such as disease diagnosis and degree of fertilisation needed (Fig. 7g–h). In the mature R8 stage, plant length and plant width significantly shrunk because of the influence of plant leaves, whereas plant height and canopy height only slightly changed (Fig. 7i).

Figure 8 shows the variation of different phenotypic parameters of a single variety during the whole growth period. According to Fig. 8, the growth periods corresponding to the maximum values of plant length, plant width, canopy height, and plant height of DN251 were R7, R7, R6 and R7, respectively. The growth periods corresponding to the maximum values of plant length, plant width, canopy height, and plant height of DN252 were R6, R5, R6, and R6, respectively. The growth periods corresponding to the maximum values of plant length, plant width, canopy height, and plant height of DN253 were R7, R6, R6, and R6, respectively. However, the growth periods corresponding to the maximum values of plant length, plant width, canopy height, and plant height of HN48 were R7, R7, R5, and R5, respectively. The growth periods corresponding to the maximum values of plant length, plant width, canopy height, and plant height of HN51 were R7, R7, R6, and R6, respectively. Therefore, the above results can be used to analyse the differences among different varieties and the phenotypic changes of the same variety in different growth stages. The results also provide timing information for field management and important phenotypic extraction.

#### Dynamic change analysis of plant growth based on 3D models

**Figure 9:**
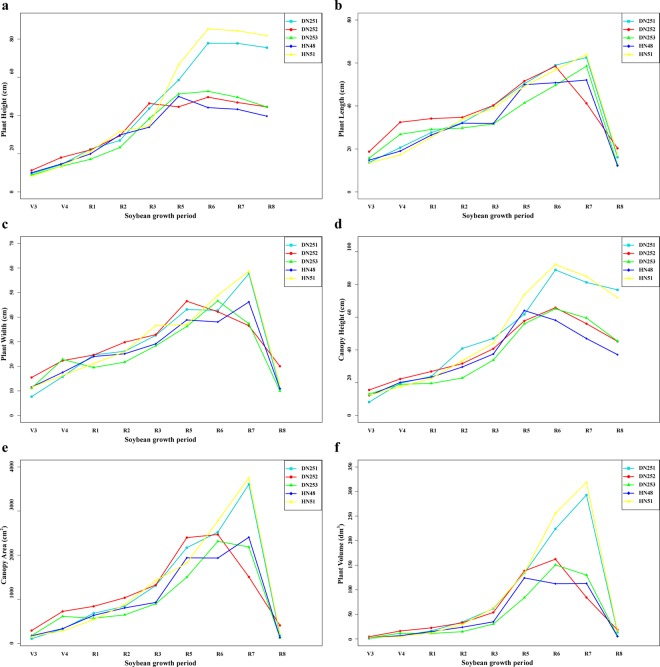
Line charts for different phenotypic traits of soybean plants. Each sub-graph shows changes of a specific trait for each of the five soybean varieties (DN251, DN252, DN253, HN48, and HN51). The phenotypes include plant height (**a**), plant length (**b**), plant width (**c**), canopy height (**d**), canopy area (**e**), and plant volume (**f**).

The dynamic changes of plant height, plant length, plant width, canopy height, canopy area, and plant volume of different soybean varieties during the whole growth period are shown in Fig. 9. At the early stages of growth, all phenotypes showed a significant upward trend. When the plant hit maturity, the plant height and canopy height slightly decreased. According to the division standard of growth periods of soybean plants^55^, the time interval of phenotypic determination in different growth periods is long. Based on the measurement standards shown in Fig. 2e,f, the plant phenotype parameters were closely related to plant leaves. As plants grew and the leaves fell off, there was a significant declining trend for the values of these four traits after reaching maturity.

However, the dynamic changes of canopy area and plant volume can also be obtained in the line chart. The maximum growth period of canopy area and plant volume of DN251 and HN51 was R7. The maximum growth period of canopy area and plant volume of DN252 and DN253 was R6. The maximum growth periods of canopy area and plant volume of HN48 were R7 and R5, respectively (Table 1).

**Table 1:** Statistics of the maximum growth period of corresponding phenotypes of different varieties.

Variety	Plant Length	Plant Width	Canopy Height	Plant Height	Canopy Area	Plant Volume
DN251	R7	R7	R6	R7	R7	R7
DN252	R6	R5	R6	R6	R6	R6
DN253	R7	R6	R6	R6	R6	R6
HN48	R7	R7	R5	R5	R7	R5
HN51	R7	R7	R6	R7	R7	R7

#### Analysis of plant logistic growth curve based on 3D models

**Figure 10:**
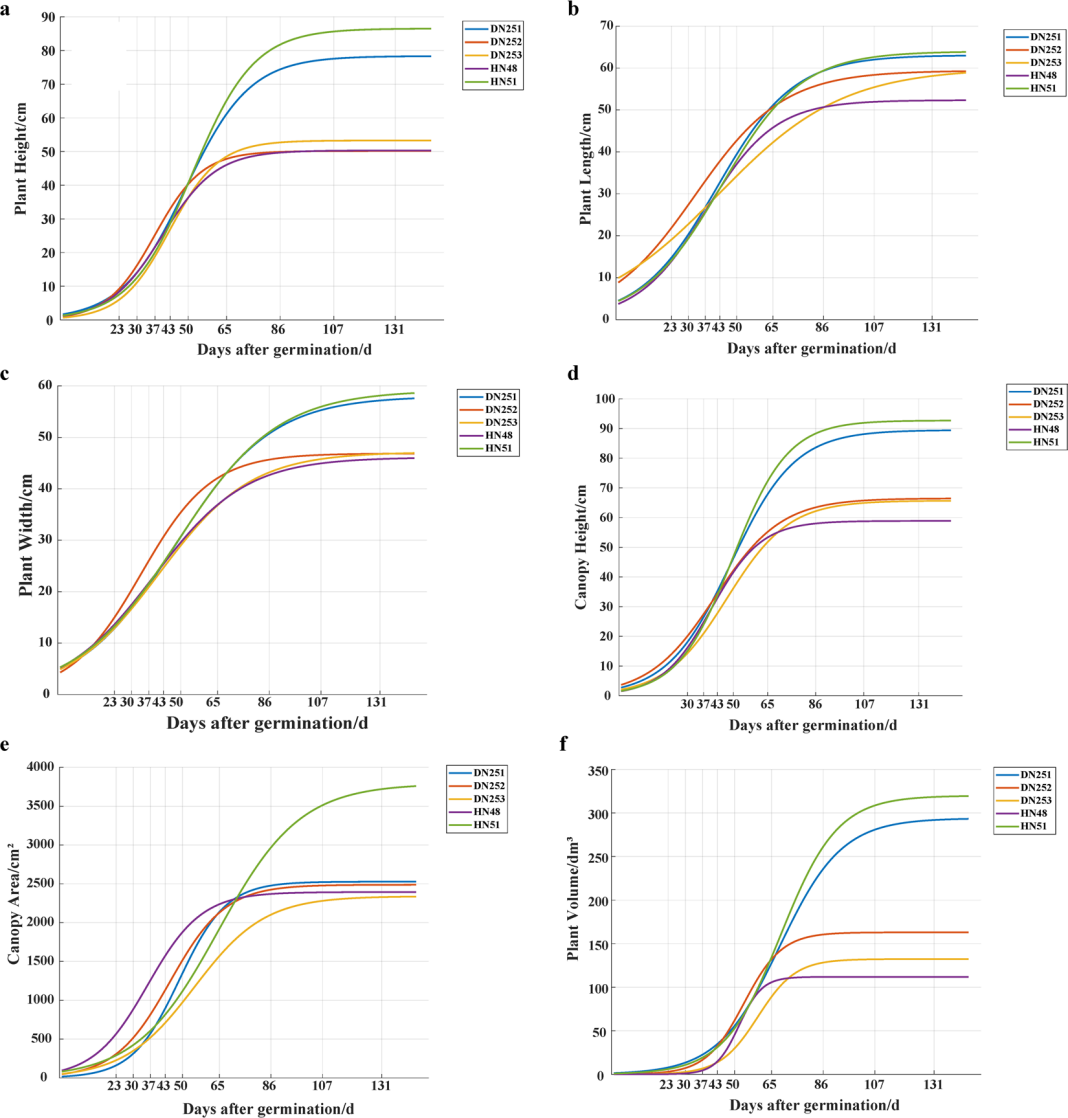
Logistic growth curves for different phenotypic traits of soybean plants. Each sub-graph shows changes of logistic growth curves of a single trait for all five soybean varieties (DN251, DN252, DN253, HN48, and HN51). The phenotypic traits include plant height (**a**), plant length (**b**), plant width (**c**), canopy height (**d**), canopy area (**e**), and plant volume (**f**).

Figure 10 shows the logistic growth curve of soybean plant phenotypes during the whole growth period based on 3D models. For plant height, the time it took for the five varieties to reach the highest growth point differed. DN252, DN253, and HN48 all reached their highest values (49.628 cm, 52.681 cm, and 49.994 cm, respectively) about 86 days after seedling emergence. DN251 and HN51 reached their highest values (77.814 cm and 85.290 cm, respectively) about 107 days after seedling emergence (Fig. 10a). For plant length, all five varieties (DN251, DN252, DN253, HN48, and HN51) reached their highest values (62.528 cm, 58.466 cm, 58.511 cm, 52.075 cm, and 63.948 cm, respectively) about 131 days after seedling emergence. The variation in plant length was similar between DN251 and HN51 (Fig. 10b).

The changes in plant width of DN251 and HN51 were the most similar among the five cultivars, and the time required for the two cultivars to reach the highest value was longer than that of the other three cultivars. The maximum plant width values of DN251, DN252, DN253, HN48, and HN51 were 57.668 cm, 46.502 cm, 46.618 cm, 46.197 cm, and 58.703 cm, respectively (Fig. 10c). For canopy height, the change trend of canopy height was similar to that of plant height. The five varieties all reached their highest growth point around 107 days after seedling emergence. The maximum canopy height values of the five cultivars (DN251, DN252, DN253, HN48, and HN51) were 88.856 cm, 65.799 cm, 65.104 cm, 64.036 cm, and 92.118 cm, respectively (Fig. 10d).

Because canopy area and plant volume are affected by plant length and plant width during the growth period, the dynamic change of canopy area and plant volume is a comprehensive result. Compared with the other four varieties, the canopy area of HN51 substantially differed, and the highest value of canopy area reached 3753 cm^2^. The maximum canopy area of DN251, DN252, DN253, and HN48 did not significantly differ (3605.848 cm^2^, 2467.031 cm^2^, 2313.884 cm^2^, and 2405.695 cm^2^, respectively; Fig. 10e). However, the differences in plant volume among the five varieties were obvious. The peak time points of DN252, DN253, and HN48 were earlier than those of DN251 and HN51. Moreover, the maximum values of DN252, DN253, and HN48 were 162.328 dm^3^, 150.643 dm^3^, and 124.156 dm^3^, respectively. The maximum plant volume values of DN251 and HN51 (292.840 dm^3^ and 318.713 dm^3^, respectively) were significantly different from those of the other three varieties (Fig. 10f). In conclusion, the logistic growth curve fitting based on the quantitative phenotypic parameters of the 3D models can clearly reveal the differences in phenotypes among different varieties.

**Figure 11:**
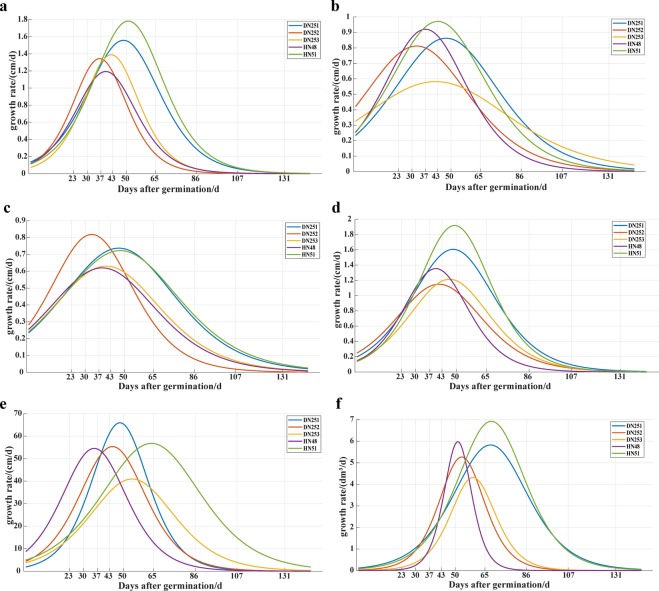
Growth rate curves for different phenotypic traits of soybean plants. Each sub-graph shows changes of growth rate curves of a single phenotypic for all five soybean varieties (DN251, DN252, DN253, HN48, and HN51). The phenotypes include plant height (**a**), plant length (**b**), plant width (**c**), canopy height (**d**), canopy area (**e**), and plant volume (**f**).

Figure 11 shows the growth rate curves of soybean plant phenotypes during the whole growth period. For plant height, the growth rate of DN252 was almost always higher than that of the other four varieties about 30 days after seedling emergence. About 37 days after emergence, the growth rate of plant height reached its maximum. The growth rate of HN51 was close to that of DN251, DN253, and HN48 in the early stage. However, the growth rate of HN51 was significantly higher than that of the other four varieties from about 32 days after emergence, and the growth rate of HN51 reached the highest value at about 52 days. The time for HN51 and DN251 to reach the maximum growth rate was similar. This is an important finding for field management of different varieties (Fig. 11a). For plant length, the growth rates of the five cultivars greatly differed. However, the five cultivars reached the maximum growth rate within 33–50 days after seedling emergence. The maximum growth rate of plant length of HN51 was higher than that of the other four varieties. The maximum growth rate of DN253 was the lowest, and there were substantial differences between DN253 and the other four varieties (Fig. 11b). For plant width, the growth rate of DN252 was higher than that of the other four varieties within 43 days after seedling emergence and reached the highest growth rate at around 34 days. The growth rates of the other four cultivars were similar within 27 days after seedling emergence; after this time point, the growth rates of DN251 and HN51 greatly increased, and reached the highest growth rate in about 48 days. However, DN253 and HN48 also reached the highest growth rate at about 40 days after seedling emergence (Fig. 11c).

For canopy height, the five varieties (DN251, DN252, DN253, HN48, and HN51) reached the highest growth rate at 49, 43, 47, 40, and 50 days after seedling emergence, respectively. The growth rate of canopy height of HN51 was also higher than that of the other four cultivars (Fig. 11d). For canopy area, DN251 and DN252 reached the maximum growth rate at approximately 48 and 45 days, respectively. HN48 was the first to reach the highest growth rate at about 35 days after seedling emergence, whereas HN51 reached the highest growth rate at about 65 days after seedling emergence (Fig. 11e). For plant volume, the growth rate of early plant volume was lower, because it was affected by multi-phenotype interactions. DN251, DN252, DN253, HN48, and HN51 reached their highest growths rate at around 68, 54, 60, 50, and 69 days after seedling emergence, respectively (Fig. 11f).

In conclusion, the phenotypic growth rates of DN251 and HN51 were similar. The growth rate differences between different phenotypes also indicated differences in morphological changes, such as differences in effective photosynthetic area and plant volume, among varieties during the whole growth period. Analysing the phenotypic parameters extracted from the 3D models provides unique insight that can be used for precision agriculture of different varieties.

## Discussion

With the continuous development of computer graphics and 3D reconstruction technology, research on 3D reconstruction of various crops has also made great progress^56^. However, because of the complexity of soybean plant types, research on the 3D reconstruction of soybean plants at home and abroad has mainly focused on the 3D reconstruction of soybean plants at the seedling stage^57^ and of their leaves^58^. In the seedling stage, there are few soybean plant leaves and the growth structure is simple. Therefore, 3D reconstruction of plants at the seedling stage is easier to accomplish. In this study, our low-cost 3D reconstruction technology successfully achieved 3D morphological reconstruction of soybean plants throughout their life. Moreover, the morphological changes of soybean plants and extraction of plant phenotypic parameters during the whole growth period were completed based on the 3D models. The 3D reconstruction models both record the morphological structure of a soybean plant during the critical periods of its life and are non-destructive. This technology provides a foundation that breeders can use to conduct virtual breeding based on forecasted future meteorological conditions.

Currently, the 3D reconstruction of most field crops is limited to the analysis of population characteristics, which is of great significance to the analysis of population-scale crop information^59–61^. However, population analysis cannot accurately extract crop phenotypes and has little significance for quantitative analysis of the morphological changes of specific phenotypes. In this paper, the proposed low-cost crop 3D reconstruction technology can indirectly facilitate plant reconstruction and phenotype parameter extraction in the field, and the growth of field plants was described and analysed through barrel plants in the field.

The 3D models obtained by 3D reconstruction can permanently preserve the plant morphology of specific varieties in a specific environment, which provides theoretical and data support for the design breeding mode, which combines molecular breeding with phenotypic breeding.

In the field of crop 3D reconstruction, precision 3D point clouds, as an important data set for analysis, are crucial for successful extraction of crop morphological features^62–64^. 3D point clouds have been obtained by many methods, such as laser radars^65^, ultrasonic sensors^66^, scanners^22^, and digitisers^67^. Compared with these technologies, the proposed method for 3D reconstruction is more economical and efficient, and more realistically restores the colour and morphological structure of plants. Therefore, this method is better able to extract quantitative characteristic parameters of soybean plants. Accurate and effective characteristic parameters, such as LAI, leaf inclination, and stem diameter, were even obtained for soybean plants with severe leaf occlusion in the R5 and R6 stages. In the future, more morphological parameters should be extracted. These parameters can also be extracted from point cloud data with similar accuracy.

In the process of crop growth, plant structure greatly influences yield when the photosynthetic and non-photosynthetic systems of the population reach relative stability^68^. To improve crop yield, it is necessary to achieve the most stable state between environmental factors and plant structure. To date, most of the research data on the selection of crop plant types were manually obtained^69^. Therefore, the extraction of plant parameters based on 3D models and deep learning can effectively avoid the damage caused by manual measurement. Plant type selection models can be divided into classification and clustering models. The classification selection model selects plant type using classification criteria^70^, whereas the cluster selection model selects phenotypic characters with obvious differences among multiple lines as the alternative phenotypic types. Field strain investigation can then be carried out according to these selected phenotypes. Subsequently, the selection of phenotypes can be completed by measuring the differences between phenotypes or hierarchical clustering analysis.

Similarly, the problem of food shortage caused by the rapidly increasing human population is becoming increasingly serious. It has become a great challenge for modern agriculture to improve crop yield through traditional crop management approaches. The key to increasing yield is to combine the extraction of precise crop phenotypes with traditional management. Knowledge regarding precise phenotypes of soybean at specific growth stages, such as plant height, plant length, plant width, plant volume, and canopy area, can help facilitate optimal management of agriculture, such as by optimising planting density according to plant volume. The combination of crop 3D models and planting density can undoubtedly help provide novel approaches to optimal management of agriculture. With the continuous development of high-throughput plant phenotypic analysis technology, more plant phenotypic information will inevitably be obtained and can be integrated to improve crop management.

## Conclusion

With the development of “precision farming”, the low-cost 3D reconstruction technology proposed in this study can be used to reconstruct the whole growth period of soybean plants. The 3D reconstruction models can show the morphological changes of soybean plants during the whole growth period. Furthermore, several phenotypic parameters, including plant height, plant length, plant width, canopy height, canopy area, and plant volume, were extracted based on the 3D models. The Pearson correlation coefficients between the phenotypic parameter values extracted from the 3D models and the manually measured values were higher than 0.98. Therefore, it can be concluded that the reconstruction accuracy of the platform was high. Based on the 3D models, growth model development, growth rate analysis, and “phenotypic fingerprint” analysis of different soybean varieties during the whole growth period were completed. The phenotypic fingerprint comprehensively showed variation of different phenotypes among varieties during the whole growth period. According to the logistic growth model of soybean plants, the corresponding time points of the maximum growth rate of the five soybean varieties were determined. These findings both provide technical and theoretical support for the design breeding mode that combines genotypic and phenotypic information, and also provides effective guidance that can enhance crop breeding and growth management. Moreover, this method provided a 3D reconstruction and parameter extraction during the whole growth period of soybean plants, but can also be applied to other crops, such as corn and wheat.

## Supplementary information

Supplementary information.

Supplementary information2.

## References

[1] Zhao CJ, Lu SL, Guo XY, Xiao BX, Wen WL (2010). Exploration of digital plant and its technology system. Scientia Agricultura Sinica.

[2] Paulus S (2014). Low-Cost 3D Systems: Suitable Tools for Plant Phenotyping. Sensors.

[3] Zhang Y, Teng P, Shimizu Y, Hosoi F, Omasa K (2016). Estimating 3D leaf and stem shape of nursery paprika plants by a novel multi-camera photography system. Sensors.

[4] Burgess, A. J., Retkute, R., Pound, M. P., Mayes, S. & Murchie, E. H. Image-based 3d canopy reconstruction to determine potential productivity in complex multi-species crop systems. *Annals of Botany* (2017). 10.1093/aob/mcw242 PMC5458713 28065926

[5] Měch, R. & Przemyslaw P. Visual models of plants interacting with their environment//ACM (1996).

[6] Fang H, Hu LC, He RT, He Y (2012). Research on plant three-dimensional information acquisition method. Transactions of the CSAE.

[7] Apelt F, Breuer D, Nikoloski Z, Stitt M, Kragler F (2015). Phytotyping4d: a light‐field imaging system for non‐invasive and accurate monitoring of spatio‐temporal plant growth. Plant Journal.

[8] Rose JC, Paulus S, Kuhlmann H (2015). Accuracy analysis of a multi-view stereo approach for phenotyping of tomato plants at the organ level. Sensors.

[9] Wang, F. *et al*. High-throughput volumetric reconstruction for 3D wheat plant architecture studies. *J Innov Opt Heal Sci*, (2016).

[10] Wither J, Frédéric B, Cani MP, Godin C (2010). Structure from silhouettes: a new paradigm for fast sketch-based design of trees. Computer Graphics Forum.

[11] Deussen, O. Digital design of nature-computer generated plants and organics//digital design of nature: computer generated plants and organics. Springer-Verlag (2015).

[12] Zhu X, Jin X, You L (2015). High-quality tree structures modelling using local convolution surface approximation. Visual Computer.

[13] Hu, B. G., Reffye, P. D., Zhao, X., Yan, H. P. & Kang, M. Z. GreenLab: A New Methodology Towards Plant Functional-Structural Model–Structural Part. International Symposium on Plant Growth Modeling, Simulation, Visualization and Their Application (2007).

[14] Vos, J., Marcelis, L. F. M., Visser, P. H. B. D., Struik, P. C. & Evers, J. B. Functional-structural plant modelling in crop production. Springer Publishing Company, Incorporated (2007).

[15] Ziegler, V. *et al*. Effects of temperature and moisture during semi-hermetic storage on the quality evaluation parameters of soybean grain and oil. Semina Ciências Agrárias (2016).

[16] Mullan DJ, Reynolds MP (2010). Quantifying genetic effects of ground cover on soil water evaporation using digital imaging. Functional Plant Biology.

[17] Xu, S. Y. Study on the key techniques for the plant architecture 3D scanner. Hua Zhong University of Science and Technology (2012).

[18] Liu G, Si YS, Feng J (2014). 3D reconstruction of agriculture and forestry crops. Transactions of the Chinese Society of Agricultural Machinery.

[19] Ivanov N, Boissard P, Chapron M, Andrieu B (1995). Computer stereo plotting for 3-d reconstruction of a maize canopy. Agricultural & Forest Meteorology.

[20] Mizuno, S., Noda, K., Ezaki, N., Takizawa, H., & Yamamoto, S. Detection of wilt by analyzing color and stereo vision data of plant// international conference on computer vision/computer graphics collaboration techniques. Springer-Verlag, 400-411 (2007).

[21] Li L, Zhang Q, Huang D (2014). A review of imaging techniques for plant phenotyping. Sensors.

[22] Kempthorne DM (2015). Surface reconstruction of wheat leaf morphology from three-dimensional scanned data. Functional Plant Biology.

[23] He, L., Y. *et al*. 3D reconstruction of Chinese hickory trees for mechanical harvest// Asabe International Meeting (2012).

[24] Sanchez-Rodriguez, J-P, Aceves-Lopez, & Alejandro. A survey on stereo vision-based autonomous navigation for multi-rotor MUAVs. *Robotica*, (2018).

[25] Duan T (2016). Dynamic quantification of canopy structure to characterize early plant vigour in wheat genotypes. Journal of Experimental Botany.

[26] Lei T, Udupa J (2003). Performance evaluation of finite normal mixture model-based image segmentation techniques[J]. IEEE Transactions on Image Processing A Publication of the IEEE Signal Processing Society.

[27] Chang SG, Yu B, Vetterli M (2000). Adaptive wavelet thresholding for image denoising and compression[J]. IEEE Transactions on Image Processing.

[28] Smith, A. R. & Blinn, J. F. Blue Screen Matting”[C]// Proceedings of the 23rd annual conference on Computer graphics and interactive techniques. DBLP, (1996).

[29] Baumberg, A., Lyons, A. & Taylor, R. 3D S.O.M.: a commercial software solution to 3D scanning. *Academic Press Professional* (2005).

[30] Fang W, Feng H, Yang W-N, Liu Q (2016). A fast 3D Reconstruction for wheat plant architecture studies in phenotyping. Journal of Agricultural Science and Technology.

[31] Weng J (1992). Camera calibration with distortion models and accuracy evaluation. Pattern Analysis & Machine Intelligence IEEE Transactions on.

[32] Salvi J, Armangué X, Batlle J (2002). A comparative review of camera calibrating methods with accuracy evaluation. Pattern Recognition.

[33] Weng, J., Cohen, P. & Herniou, M. Calibration of stereo cameras using a non-linear distortion model [CCD sensory]. International Conference on Pattern Recognition. IEEE Xplore (1990).

[34] Tsai RY (2003). A versatile camera calibration technique for high-accuracy 3d machine vision metrology using off-the-shelf tv cameras and lenses. IEEE Journal on Robotics & Automation.

[35] Fischler, M. A. Readings in computer vision || random sample consensus: a paradigm for model fitting with applications to image analysis and automated cartography. Readings in Computer Vision, 726-740 (1987).

[36] Wan X, Xu G (1996). Camera parameters estimation and evaluation in active vision system. Pattern Recognition.

[37] Wu Y, Li Y, Hu Z (2008). Detecting and handling unreliable points for camera parameter estimation. International Journal of Computer Vision.

[38] Scaramuzza D (2011). 1-point-ransac structure from motion for vehicle-mounted cameras by exploiting non-holonomic constraints. International Journal of Computer Vision.

[39] Naroditsky O, Zhou XS, Gallier J, Roumeliotis SI, Daniilidis K (2012). Two efficient solutions for visual odometry using directional correspondence. IEEE Transactions on Pattern Analysis & Machine Intelligence.

[40] Chen CS, Hung YP, Cheng JB (2002). Ransac-based darces: a new approach to fast automatic registration of partially overlapping range images. IEEE Transactions on Pattern Analysis & Machine Intelligence.

[41] González-Aguilera D, Rodríguez-Gonzálvez P, Hernández-López D, Lerma JL (2012). A robust and hierarchical approach for the automatic co-registration of intensity and visible images. Optics and Laser Technology.

[42] Kim JH, Han JH (2006). Outlier correction from uncalibrated image sequence using the triangulation method. Pattern Recognition.

[43] Mai F, Hung Y, Zhong H, Sze W (2008). A hierarchical approach for fast and robust ellipse extraction. Pattern Recognition.

[44] Lv YW, Feng JL, Li ZK, Liu W, Cao JT (2015). A new robust 2D camera calibration method using RANSAC. Optik - International Journal for Light and Electron Optics.

[45] Zhou F, Cui Y, Wang Y, Liu L, Gao H (2013). Accurate and robust estimation of camera parameters using RANSAC. Optics & Lasers in Engineering.

[46] Deng, Y.Z. Research on technology of computer 3D reconstruction based on image. Xi’an University of Architecture and Technology (2011).

[47] Zhang, K. Research on 3D Surface Reconstruction Algorithm Based on Poisson Equation. *Hebei University of Technology* (2014).

[48] Sun, K. Research on the optimization of planting density based on 3D reconstruction for soybean planted by PuLan seed company. *Northeast Agricultural University* (2019).

[49] Yao WQ, Zheng JL, Chen P, Chen WN (2016). An Octree-based mesh simplification algorithms for 3-dimension cloud data. Science of Surveying and Mapping.

[50] Liu B, Guo BM, Deng XX (2016). A point cloud registration method based on Octree and ICP. Science of Surveying and Mapping.

[51] Wang M, Feng JQ, Yang B (2014). Comparison and evaluation of marching cubes and marching tetrahedra. Journal of Computer-Aided Design & Computer Graphics..

[52] Qiu, L.J. Descriptors and data standard for soybean (glycine spp.) 2-6. China Agriculture Press (2006).

[53] Harris D (1989). NCME Instructional Module: Comparison of One-. Two-, and Three-Parameter IRT Models[J]..

[54] Raschka S. Python Machine Learning. (Packt Publishing, 2015).

[55] Pedersen P. et al. Soybean growth and development. (Ames, IA: Iowa State University, University Extension, 2004).

[56] Liu G, Si YS, Feng J (2014). 3D reconstruction of agriculture and forestry crops. Transactions of the Chinese Society for Agricultural Machinery.

[57] Song QP, Tang JL, Xin J (2017). 3-dimensional reconstruction for soybean plant of seedling stage based on growth model. Computer Engineering.

[58] Xie QJ, Su ZB, Sun HM (2011). Research on technology for soybean leaf 3D reconstruction and deformation modeling. Journal of Agricultural Mechanization Research.

[59] Jay S, Rabatel G, Hadoux X, Moura D, Gorretta N (2015). In-field crop row phenotyping from 3d modeling performed using structure from motion. Computers and Electronics in Agriculture.

[60] Biskup B, Scharr H, Schurr U, Rascher U (2010). A stereo imaging system for measuring structural parameters of plant canopies. Plant Cell & Environment.

[61] Ali S, Suhas K, Felix F, Guilherme DS (2017). Vinobot and vinoculer: two robotic platforms for high-throughput field phenotyping. Sensors.

[62] Anthony P, Xavier S, Scott B, Robert F, Jurgen F (2012). A novel mesh processing based technique for 3D plant analysis. BMC Plant Biology.

[63] Paulus S, Dupuis J, Mahlein AK, Kuhlmann H (2013). Surface feature based classification of plant organs from 3D laserscanned point clouds for plant phenotyping. BMC Bioinformatics.

[64] Paulus S, Schumann H, Kuhlmann H, Jens Léon (2014). High-precision laser scanning system for capturing 3d plant architecture and analysing growth of cereal plants. Biosystems Engineering.

[65] Sun SP (2018). In-field high throughput phenotyping and cotton plant growth analysis using lidar. Frontiers in Plant Science.

[66] Krammer P, Schweinzer H (2006). Localization of object edges in arbitrary spatial positions based on ultrasonic data. IEEE Sensors Journal.

[67] Wen W, Guo X, Wang Y, Li C, Lu S (2015). Morphological and structural data acquisition for above-ground part of grapevine. Transactions of the Chinese Society of Agricultural Engineering.

[68] Wang, Q. C., Niu, Y. Z., Xu, Q. Z., Wang, Z. X., & Zhang, J. Effect of Plant-type on Rate of Canopy Apparent Photosynthesis and Yield in Maize (Zea mays L.). *Acta Agronomica S*inica, **(**02**)**:97–101 (1996).

[69] Tang, J. H. *et al*. Effects of tillage patterns on spatial distribution of seeds and yield of summer soybean in north Xinjiang. *Agricultural Research in the Arid Areas* (2015).

[70] You, M. A. A preliminary study on soybean yield distribution in space. *Soybean Science* (1993).

